# Nesogordonia tricarpellata (Dombeyaceae), a new species from Madagascar that compels modification of the morphological circumscription of the genus

**DOI:** 10.3897/PhytoKeys.2.747

**Published:** 2011-02-11

**Authors:** Cynthia Skema, Laurence J. Dorr

**Affiliations:** 1 Institute of Molecular BioSciences, Massey University, Private Bag 11222, Palmerston North 4442, New Zealand; 2 Department of Botany, MRC-166, National Museum of Natural History, Smithsonian Institution, P.O. Box 37012, Washington, DC 20013-7012 U.S.A.

**Keywords:** Anosyenne Mountains, conservation, Dombeyaceae, endemism, Madagascar, Malvaceae, *Nesogordonia*, taxonomy

## Abstract

Nesogordonia tricarpellata Skema & Dorr, **sp. nov.**, a new species from southeastern Madagascar, is described and illustrated. It differs from all other species of Nesogordonia Baill. in having 6–9 stamens, 3 staminodes, a 3-carpellate ovary, and a 3-valved capsule. These androecial and gynoecial characters require modification of the long-standing circumscription of the genus. The new species also has the southernmost geographic range of any species in the genus.

## Introduction

Nesogordonia Baill. (Dombeyaceae, or Malvaceae: Dombeyoideae) is a genus of ca. 20 species of small to large trees (Arènes 1959, Barnett 1988, Dorr and Barnett in Cheek and Dorr 2007). Three species occur in tropical Africa and one in the Comoro Islands (Labat et al. 2000). The majority of species, however, are endemic to Madagascar and as previously unexplored areas of this island are surveyed botanically additional novelties are discovered (Barnett 1987, Callmander et al. 2009, Rakotoarivelo et al. 2010), including the following new species that compels us to modify the long-established morphological circumscription of the genus.

## Systematics

### Nesogordonia tricarpellata

Skema & Dorr sp. nov.

urn:lsid:ipni.org:names:77109527-1

Fig. 1 

#### Latin

Species gynecio 3-carpello et 3 staminodiis a congeneribus differt.

#### Type.

 **Madagascar:** Toliara: Anosy, Fort-Dauphin, Iabakoho, Antsotso, forêt humide de basse altitude Ivohibe–Bemangidy, près d’un cours d’eau, 24°34'17"S, 047°12'07"E, 90 m. 12 February 2006 (fl). J. Rabenantoandro, F. Randriatafika, B. Mara, P. Lowry, and E. Lowry 1711 (holotype: US!; isotypes: MO!, TAN!).

#### Description.

*Tree*, 4–8 m tall, to 15 cm d.b.h.; young stems glabrous, pale whitish gray to brown with prominent leaf scars; older stems darker brown; terminal bud to 3 mm long, subulate to falcate, strigose. *Leaves* alternate, entire, persistent; petioles 1–1.5 cm long, glabrous or with a few minute, stellate hairs or with a row of simple hairs (Randriamampionona 470), slightly to noticeably pulvinate basally and apically, drying dark brown; blade elliptic to narrowly elliptic, 5–7.5 × 2.5–3 cm, subcoriaceous, dark green above, lighter green below, base obtuse to rounded or attenuate (Randriamampionona 451), margin shallowly crenulate, slightly revolute, apex long acuminate, mucronulate (as an extension of the 1° vein below), glabrous above and below or with mostly simple, erect to appressed hairs restricted to 1° vein above (Randriamampionona 470), venation brochidodromous, 1° vein conspicuously raised below, 2° veins visible but less prominently raised below; domatia ovoid to almost circular tufts of erect or arching simple and stellate hairs in axils of 1° and 2° veins below, 0.6–0.9 × 0.5–0.6 mm; stipules caducous, not seen. *Inflorescences* axillary, paniculate cymes, to 4.5 cm long, (1–) 2–3-flowered; peduncles to 2.6 cm long, glabrous or with a few minute, stellate hairs; pedicels to 1.5 cm long, articulated 3.2–4.3 mm below base of the flower, glabrous or with a few minute, stellate hairs especially above the articulation. *Epicalyx* fugacious, not seen. *Flower* budsglobose, 3–4 × 3–4 mm, sepals valvate, sutures slightly raised. *Calyx* 5-parted, shortly fused at base; *sepals* ovate, 5–5.5 × 1.7–2.2 mm, fleshy, heterotrichous outside with shorter-armed (ca. 0.02 mm long) stellate hairs beneath sparsely distributed longer-armed (ca. 0.2 mm long) stellate hairs, sparingly pubescent inside with few stellate hairs mostly near the sutures, both surfaces with a denser patch of minute stellate or simple hairs apically. *Petals* 5, ovate to elliptic, 3.5–4 × 2–2.5 mm, weakly asymmetric, slightly constricted apically, fleshy, glabrous, white. *Androecium* biseriate, outer whorl of 3 fascicles of 2–3 stamens each (6–9 stamens total), shortly (to 0.5 mm) fused basally, inner whorl of 3 staminodes; stamens laminar; anthers 2.7–2.8 × 0.8–1.1 mm; lanceolate to oblong, asymmetric, 2.8–2.9 × 0.8 mm, apex acute to acuminate, fleshy. *Gynoecium* superior, ca. 1.5 × 1.5 mm, 3-carpellate, densely covered by small lepidote scales (ca. 0.15 mm in diameter); ovules 2 per carpel, basally winged, placentation subapical, axile; styles 3, connate, 2–2.5 mm long; stigma lobes 3, ca. 0.8 mm long, fleshy, stigmatic surface on interior apical portion of lobes, deep red drying black. *Capsules* woody, obconic, 1.5–2 × 1.2–1.5 cm, slightly verrucose with scattered scales or short-armed stellate hairs, chestnut brown, apex depressed with a central umbo, rim not present or scarcely developed. *Seeds* 4–5 × 3–4 mm, seed wings 5–10 × 4–5 mm.

**Figure 1.**
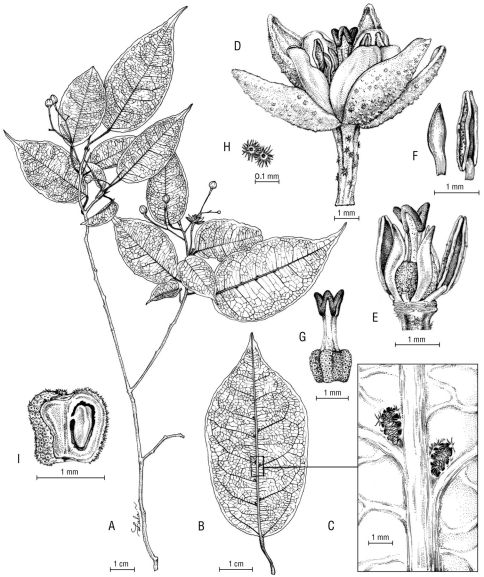
Nesogordonia tricarpellata Skema & Dorr. **A** habit **B** leaf blade below **C** leaf blade below, showing detail of domatia **D** flower **E** flower, calyx and corolla removed to show stamens, staminodes, ovary, stigma, and style **F** anther (right) and staminode (left) **G** gynoecium with lepidote scales on ovary and 3-parted style **H** detail of lepidote scales **I** immature fruit, longitudinal section showing position of winged ovule. Line drawing by L.R. Andriamiarisoa from herbarium specimen; voucher Rabenantoandro et al. 1711 (MO).

#### Distribution.

**Figure 2.**
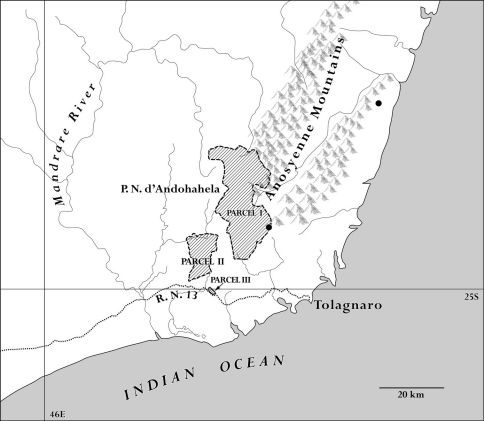
Map of extreme southeastern Madagascar showing where Nesogordonia tricarpellata Skema & Dorr has been collected and these two localities in relation to the three parcels that comprise the Parc National d’Andohahela.

Endemic to southeastern Madagascar, where it is known from two collections made in Parcelle 1 of the Parc National d’Andohahela (formerly Réserve Naturelle Intégrale d’Andohahela) and one on the lower slopes of the Ivohibe–Bemangidy forest (Fig. 2).

#### Ecology.

Evidently restricted to humid forest from 90–500 m. Flowering specimens were collected in February (floral buds) and June (mature flowers). A fruiting specimen was collected in June.

#### Etymology.

 The epithet was chosen to highlight the fact that the gynoecium of this species is 3-carpellate.

#### Conservation status.

 At present, this species is known from three collections and two localities, only one of which is protected (Parc National d’Andohahela). Based on this and estimates of an extent of occurence < 100 km2 and an area of occupancy < 10 km2, Nesogordonia tricarpellata is assigned a preliminary status of Critically Endangered (CR B1ab(i-iv) + B2ab(i-iv)) following the criteria and categories of the IUCN (2001).

#### Specimens examined.

 **Madagascar:** Toliara: Intégrale Réserve # 11, Andohahela, Parcelle 1, Isaka Ivondro, 24°40'S, 46°52'E, 100–150 m. 12–23 June 1993 (fl), Randriamampionona 451 (MO), Ibid., 12–23 June 1993 (fr), Randriamampionona 470 (MO).

#### Discussion.

 We have no doubt that this new speciesbelongs in Nesogordonia asit has the fruit and seeds unique to the genus: an obovoid woody capsule containing seeds with long, basal wings (Fig. 1I). Nesogordonia tricarpellata is remarkable in having an androecium comprised of 6–9 stamens in an outer whorl and 3 staminodes in an inner whorl; a 3-carpellate ovary; 3 style branches and 3 stigma lobes; and a 3-valved capsule (Fig. 1E, G). While the number of stamens is variable in other species of Nesogordonia, the outer whorl typically possesses some multiple of five (usually 10–25 total) stamens and the inner whorl possesses either 5 free staminodes (most species) or 5 stamens (Nesogordonia abrahamii L.C. Barnett, Nesogordonia ambalabeensis Arènes, and Nesogordonia fertilis H. Perrier). All other species of Nesogordonia have 5-carpellate ovaries, 5 style branches, 5 stigma lobes, and 5-valved capsules. Although the parts of the androecium and gynoecium of Nesogordonia tricarpellata are reduced in number, the flowers of the new species are 5-merous with 5 calyx lobes and 5 petals, which also is characteristic of all other species of Nesogordonia (Fig. 1D).

Carpel number is variable in the Dombeyaceae, and it even varies within a genus (8 of 19 genera; Bayer and Kubitzki 2003). It is not surprising therefore that carpel number in Nesogordonia also is variable and the generic description should be modified to accommodate taxa that are 3-carpellate. Among the Malagasy genera of Dombeyaceae, only Dombeya Cav. (2-, 3-, or 5-carpellate) and Helmiopsis H. Perrier (3- or 5-carpellate) also have species that are 3-carpellate (Arènes 1959, Skema and Dorr 2010). Like Nesogordonia tricarpellata, these other 3-carpellate species also possess a pentamerous perianth.

Nesogordonia tricarpellata is the only species in the genus with lepidote scales on the ovary (Fig. 1H, I). All other species of Nesogordonia have stellate hairs on the ovary (Barnett 1988). As has been noted before (Jenny et al. 1999, Dorr 2001), a number of other genera in the Dombeyaceae have species with either stellate hairs or lepidote scales on the ovary, including Dombeya, Harmsia K. Schum., and Helmiopsis.

Nesogordonia tricarpellata most closely resembles Nesogordonia micrantha Arènes. The two species have similar leaf shape, size, and vestiture; inflorescence morphology; and floral bud shape. The leaf blades of both species are glabrous to sparingly pubescent with domatia of tufted hairs in the axils of the 1° and 2° veins below. The leaves of Nesogordonia micrantha, however, are elliptic to obovate (versus elliptic to narrowly elliptic), 2.2–5 × 1.1–2.8 cm (versus 5–7.5 × 2.5–3 cm), apically acute (versus long acuminate), and the margin is undulate to slightly crenulate (versus slightly crenulate). Both species have (1–) 2–3-flowered axillary, paniculate cymes and globose floral buds. The floral buds of Nesogordonia micrantha, however, are densely (versus sparingly) stellate pubescent. In addition, the two species have non-overlapping geographical ranges; Nesogordonia tricarpellata appears to be restricted to the Anosyenne Mountains in southeastern Madagascar while Nesogordonia micrantha is known only from western Madagascar having been collected principally in the Forêt d’Antsingy.

The geographical distribution of Nesogordonia tricarpellata also is remarkable as the species has the southernmost distribution of any species in the genus. Nesogordonia tricarpellata appears to be restricted to humid forest on the eastern slopes of the Anosyenne Mountains in extreme southeastern Madagascar south of the Tropic of Capricorn (Fig. 2). The Parc National d’Andohahela includes the southernmost moist “tropical” forest in Madagascar, a forest that appears to be an extension of the humid montane forest common to the north but found at lower elevations in the Anosyenne Mountains (Goodman 1999).

Sequence data for one nuclear ribosomal (ITS) and five noncoding plastid markers have been gathered for Nesogordonia tricarpellata as part of an ongoing phylogenetic study of Dombeyaceae(Skema in prep.). Parsimony analysis of these data group this new species with Nesogordonia humbertii Capuron (Randrianaivo et al. 1391), the only other species of Nesogordonia sampled, with high support (bootstrap = 100% from 10,000 replicates).

## Supplementary Material

XML Treatment for Nesogordonia tricarpellata
